# Bacterial Outer Membrane Vesicles and Immune Modulation of the Host

**DOI:** 10.3390/membranes13090752

**Published:** 2023-08-24

**Authors:** Lily A. Charpentier, Emily F. Dolben, Matthew R. Hendricks, Deborah A. Hogan, Jennifer M. Bomberger, Bruce A. Stanton

**Affiliations:** 1 Department of Microbiology and Immunology, Geisel School of Medicine at Dartmouth, Hanover, NH 03755, USA; lily.a.charpentier.gr@dartmouth.edu (L.A.C.); efdaniels@gmail.com (E.F.D.); deborah.a.hogan@dartmouth.edu (D.A.H.); jennifer.m.bomberger@dartmouth.edu (J.M.B.); 2 Department of Microbiology and Molecular Genetics, University of Pittsburgh, Pittsburgh, PA 15219, USA; matthew.hendricks@gilead.com

**Keywords:** outer membrane vesicles (OMVs), DNA methylation (DNAm), immune modulation, inter-kingdom communication

## Abstract

This article reviews the role of outer membrane vesicles (OMVs) in mediating the interaction between Gram-negative bacteria and their human hosts. OMVs are produced by a diverse range of Gram-negative bacteria during infection and play a critical role in facilitating host–pathogen interactions without requiring direct cell-to-cell contact. This article describes the mechanisms by which OMVs are formed and subsequently interact with host cells, leading to the transport of microbial protein virulence factors and short interfering RNAs (sRNA) to their host targets, exerting their immunomodulatory effects by targeting specific host signaling pathways. Specifically, this review highlights mechanisms by which OMVs facilitate chronic infection through epigenetic modification of the host immune response. Finally, this review identifies critical knowledge gaps in the field and offers potential avenues for future OMV research, specifically regarding rigor and reproducibility in OMV isolation and characterization methods.

## 1. Introduction

Gram-negative bacteria employ diverse mechanisms to interact with other bacteria and their human hosts. Among these mechanisms, outer membrane vesicles (OMVs) are critical in facilitating host–pathogen interactions without requiring direct cell-to-cell contact. This is particularly relevant when bacteria colonize the mucus that overlays host epithelial cells [1,2]. OMVs are spheroidal proteoliposomes ranging from ~20 to 200 nm in diameter that originate from the outer membrane of Gram-negative bacteria [3,4,5,6]. These vesicles contain various cytoplasmic and periplasmic components, including proteins, DNA, RNA, and metabolites [7,8,9,10]. The lipid bilayer of OMVs protects the contents from extra-vesicular proteins, such as proteases and RNases [2]. OMVs were first characterized in 1967 by Chatterjee and Das by transmission-electron microscopy of *Vibrio cholerae* [11]. Studies in the years since have shown that OMVs are produced by a diverse range of Gram-negative bacteria during infection and have been isolated from both pathogenic and commensal bacteria colonizing the human gut and lung. Although Gram-negative bacteria also release outer-inner membrane vesicles (O-IMVs) that derive from the bacterium’s inner and outer membrane, these vesicles account for less than 1% of the total secreted vesicles [12]. Both OMVs and O-IMVs encapsulate bacterial factors that modulate the host’s immune response to infection. These factors include proteins that inhibit epithelial chloride ion secretion and small interfering RNA (sRNA) that bind to and silence host mRNA transcripts. Despite the significant progress that has been made in characterizing the immunomodulatory properties of OMVs, many of their mechanisms of action remain elusive. Therefore, investigating the interplay between host and OMVs is crucial for developing novel therapies against bacterial infections and the ensuing inflammatory response. Recent reviews explore the intricate interactions between OMVs and the host immune response [13,14,15,16,17,18,19,20,21].

Despite the growing interest in OMVs as mediators of host–pathogen interactions, only a few studies have investigated the mechanisms underlying OMV-induced regulation of the host immune response to infection. OMVs have been shown to contain virulence factors such as sRNA, that like microRNAs (miRNA), target host immune cell genes to downregulate the host response to infection, thereby allowing microbes to establish chronic infections [2]. Although a few bacterial factors have been identified in OMVs that elicit a reduced immune response, little is known about their epigenetic mechanisms of action. Research has begun to delve into the epigenetic mechanisms behind bacterial interaction with host immune cells. This involves virulence factors that cause altered DNA methylation (DNAm) patterns in immune cells, resulting in a decreased immune response to subsequent infections and contributing to the establishment of chronic bacterial infections. For instance, *Pseudomonas aeruginosa* OMVs can downregulate the human macrophage immune response to infection by inducing changes in DNAm patterns [22]. Similarly, OMVs derived from the human gut commensal *Bacteroides thetaiotaomicron* reduce the inflammatory response to colitis-inducing dextran sodium sulfate in a mouse model [23]. Although some research has examined the immunomodulatory effects of OMVs, few studies have explored the underlying mechanisms of these interactions, specifically regarding the epigenetic modification of the host immune response to subsequent infections.

This review provides an overview of the current state of knowledge on how Gram-negative pathogens utilize OMVs to modulate the host immune response during infection. Specifically, this review highlights the novel mechanisms by which OMVs are formed and subsequently interact with host cells, leading to alternative methylation patterns of immune genes. Additionally, this review identifies critical knowledge gaps in the field and offers potential avenues for future research.

## 2. OMV Biogenesis

**Figure 1:**
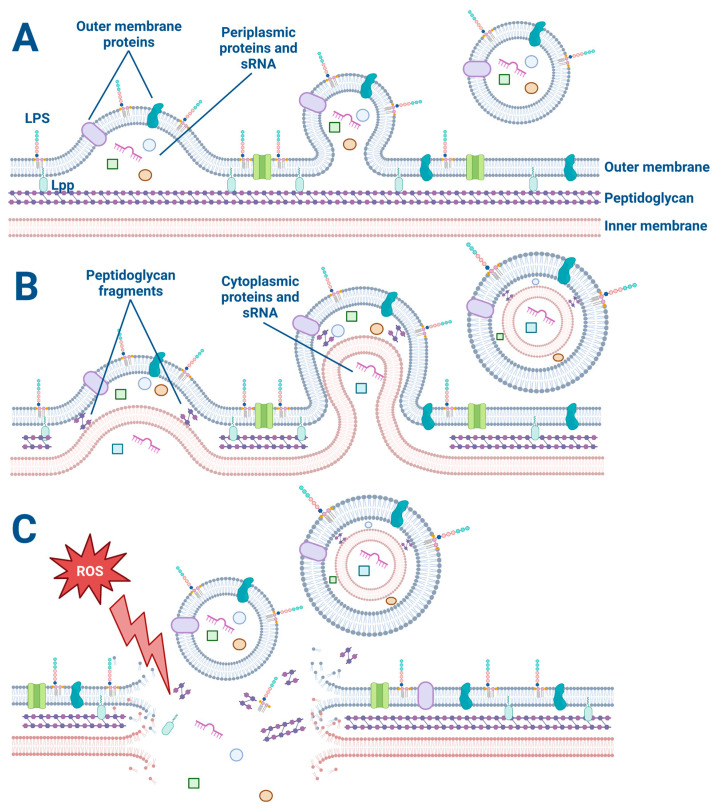
The explosive cell lysis and budding models of OMV and O-IMV generation. (**A**) The budding mechanism of OMV generation. Lipoproteins (Lpp) anchor the outer membrane to the peptidoglycan. Membrane proteins and lipopolysaccharides (LPS) decorate the outer membrane and are incorporated into the budding OMV along with periplasmic proteins and sRNA. (**B**) The budding model of O-IMV generation. Both cytoplasmic and periplasmic proteins and sRNA are incorporated into budding O-IMVs. (**C**) Cellular stress such as exposure to reactive oxygen species disrupts the membrane of a Gram-negative bacterium, causing membrane fragments to encapsulate free periplasmic and cytoplasmic material and form OMVs and O-IMVs.

The biogenesis of OMVs has been a topic of intense research in recent years due to the diverse roles that OMVs play in bacterial pathogenesis, immune modulation, and potential therapeutic applications. The explosive cell lysis and budding models are two proposed mechanisms for OMV generation in Gram-negative bacteria (Figure 1).

The explosive cell lysis model proposes that OMVs are generated as a result of a sudden and catastrophic rupture of the bacterial cell membrane. In this model, a high amount of stress on the bacterial cell can lead to a breakdown in membrane integrity and a subsequent release of large amounts of cytoplasmic and periplasmic contents, including membrane fragments [24,25]. The released membrane fragments can spontaneously assemble into OMVs. This model is supported by observations of high levels of OMVs in bacterial cultures undergoing stress or lysis, such as during antibiotic treatment or exposure to detergents [24,25,26]. The use of a live–dead staining assay on bacteria is beneficial for all OMV studies to determine if some OMVs are formed from explosive cell lysis versus another budding mechanism [27,28]. For example, preliminary studies in our laboratory have revealed that low concentrations (1 μg/mL) of the antibiotic Tobramycin kill about 10% of *P. aeruginosa* (PA14) as determined by the live–dead assay.

**Figure 2:**
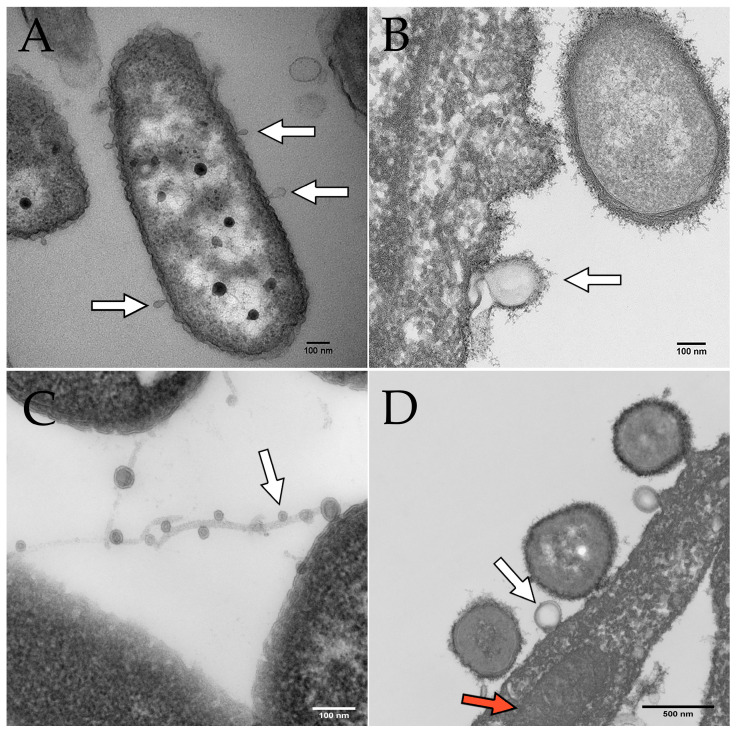
Electron microscopy images of OMVs budding from *P. aeruginosa*. Scale bars indicate 100 nm in panels (**A**–**C**) and 500 nm in panel (**D**). White arrows indicate OMVs in all panels. (**A**) OMVs budding from *P. aeruginosa* PAO1 cultured on human bronchial epithelial cells. (**B**) OMV budding from *P. aeruginosa* PA14 grown in Minimal Essential Medium (MEM) with 0.4% arginine. (**C**) OMVs on filamentous structures produced by *P. aeruginosa*. (**D**) *P. aeruginosa* OMV (derived from PAO1 grown in MEM with 10 mM glucose and 8 µM FeCl_3_) fusing with a eukaryotic cell. The red arrow indicates a mitochondrion in the airway epithelial cell.

On the other hand, the budding model proposes that OMVs are generated by a more controlled process involving the gradual formation and release of vesicles from the bacterial outer membrane. In this model, the budding of OMVs is thought to involve a selective packaging of cargo, such as proteins, lipids, and nucleic acids, into the vesicles [29]. Once the cargo is packaged, the OMVs are released from the outer membrane [29]. This model is supported by observations of asymmetrically shaped OMVs that display a more uniform size and cargo composition than those generated by explosive cell lysis [30]. Furthermore, OMV budding has been observed by electron microscopy of *P. aeruginosa* biofilms (Figure 2). In some cases, the budding of OMVs may also be triggered by bacterial stressors such as oxidative stress or exposure to antimicrobial agents [26,29].

While both the explosive cell lysis and budding models have their merits and are not mutually exclusive, it is important to note that the relative contribution of each process to OMV biogenesis may depend on the bacterial species and environmental conditions. Some studies have suggested that most OMVs are generated by the budding model [29,30,31], while others have proposed that both mechanisms can contribute equally to OMV biogenesis [32].

Further research is needed to fully understand the mechanisms involved in OMV biogenesis, how RNA, DNA, and other virulence factors are differentially packaged in OMVs, and to develop strategies for engineering OMVs with specific cargo and properties for various applications, including the development of novel treatments against chronic bacterial infections.

## 3. OMV Factors Modulate the Host Immune Response to Infection

Upon release by microbes, whether through explosive cell lysis or budding, OMVs diffuse through their environment, including mucus, to deliver their contents to recipient cells. OMVs have been shown to fuse with lipid rafts located on the membranes of epithelial cells, and to be taken up by host cells by phagocytosis or through other mechanisms such as clathrin-dependent endocytosis, caveolin-mediated endocytosis, and membrane fusion [29,33,34]. OMVs contain various types of cargo, including proteins, sRNA, and transfer RNA fragments (tRNA-fragments), which target host immune genes. Some OMV proteins, for instance, have been shown to upregulate the host immune response by stimulating host Toll-like receptors (TLRs) [35,36,37,38,39]. OMV sRNA and tRNA fragments, similar to eukaryotic miRNAs, can decrease host mRNA transcript stability, as well as regulate transcription and translation of target genes [10]. Suppression of the host immune response is advantageous for establishing and maintaining bacterial infections. Studies are encouraged to elucidate novel factors within OMVs that specifically modulate the human immune response to infection, with the expectation that this may lead to the development of novel therapeutics to fight bacterial infections and inflammation.

During infection, OMVs provide a valuable mechanism for transporting microbial virulence factors to their host targets, particularly in diseases characterized by chronic infections or colonization of mucus layers residing above epithelia. For example, OMVs from pathogenic bacteria such as *P. aeruginosa* contain flagellin and LPS, which can activate the host immune system by stimulating TLR5 and TLR4, respectively [38,39]. Recognition of the lipidA portion of LPS by TLR4 [39] leads to a signaling cascade through MyD88 and NF-κB, increases the production of hyperinflammatory cytokines such as IL-8, which in turn recruits immune cells to the lungs in an attempt to eliminate infection [40]. Some OMV proteins can exert their immunomodulatory effects by targeting specific host signaling pathways. For example, OMVs from *Burkholderia pseudomallei* contain the effector protein BopE, which can bind to the host GTPase Rac1 and activate downstream signaling events, promoting bacterial invasion and intracellular survival [41,42]. The immunomodulatory proteins found in bacterial OMVs represent an important mechanism by which bacteria can manipulate the host immune response to their advantage.

*P. aeruginosa* OMVs also carry Cif (CFTR Inhibitory Factor), a protein that interferes with the endocytic cycling of the cystic fibrosis (CF) transmembrane conductance regulator (CFTR) chloride ion channel in host epithelial cells [43,44,45,46,47,48,49,50,51,52,53]. Cif disrupts ion transport across epithelial cell membranes by promoting CFTR degradation and leads to dehydration of the airway surface layer and decreased mucociliary clearance of invading pathogens [50,54]. Cif has also been shown to prevent Major Histocompatibility Complex antigen presentation and CD8 T cell killing [55]. Similarly, delivery of the toxin CNF1 by *Escherichia coli* OMVs impairs neutrophil chemotaxis [56]. On the other hand, *Helicobacter pylori* OMVs harbor the CagA oncoprotein that is translocated into host cells and alters ATP affinity for the H1 histone, leading to an increase in DNA binding, cellular transformation, and oncogenesis [57,58,59]. OMVs can also contain proteases that cleave and inactivate host antimicrobial peptides and enzymes that degrade host extracellular matrix components, facilitating bacterial dissemination [60,61]. Bacterial OMV immunomodulatory proteins are an essential mechanism for bacteria to manipulate the host immune response. To combat bacterial infections and prevent antibiotic resistance emergence, it is crucial to understand the intricate interplay between OMV proteins and the host immune system.

OMVs also contain sRNA and tRNA fragments (~35 nt) important for modulating host gene expression by binding to mRNA transcripts, affecting translation and transcript stability [10]. For example, Choi et al. has shown that sRNAs in OMVs secreted by *Aggregatibacter actinomycetemcomitans*, *Porphyromonas gingivalis*, and *Treponema denticola* decrease cytokine secretion by Jurkat T cells, thus suppressing the immune response [62]. More examples of OMV sRNA-host interaction have been reviewed in detail [20].

One of the most striking findings is that tRNA fragments from bacterial OMVs can target and decrease the expression of host immune genes. For example, the *P. aeruginosa* methionine tRNA fragment sRNA52320 secreted in OMVs targets multiple kinases in the LPS-stimulated MAPK signaling pathway, decreasing IL-8 secretion of human bronchial epithelial cells and downregulating neutrophil recruitment in a mouse lung infection model [2]. The mechanisms by which tRNA fragments from bacterial OMVs target host mRNA transcripts are not yet fully understood. It is thought that the tRNA halves may act as decoys that compete with host miRNAs for binding to target mRNA transcripts or may interact directly with target transcripts through complementary base pairing, possibly by interacting with the AGO2/RISC complex, thereby using the host miRNA mechanism to pair with target mRNA [63,64,65,66,67,68]. However, at the present time, the mechanism whereby bacterial sRNAs and tRNAs inhibit gene expression in eukaryotic hosts is incompletely understood and, therefore, warrants study.

In summary, OMV-derived proteins and RNA fragments play a crucial role in reducing the host immune response. The identification and characterization of additional novel factors within OMVs that specifically modulate the human immune response to infection may lead to the development of novel therapeutics to fight chronic bacterial infections and prevent the emergence of antibiotic resistance. 

## 4. OMVs Alter the Host Immune Response to Subsequent Bacterial Infections through Alternative Methylation of Immune Genes

Trained immunity is a concept that challenges the traditional view of the immune system as a static, pre-programmed system. Instead, trained immunity suggests that the immune system can be trained or “primed” to provide enhanced protection against subsequent infections [69,70,71]. This phenomenon is mediated by immune cells such as macrophages and natural killer cells, which can be reprogrammed by exposure to certain stimuli to produce either a more or less robust and efficient immune response [71]. Early-in-life infections in people with CF are typically cleared with antibiotics through several cycles before eventually establishing a chronic infection [72,73,74]. Compared to non-CF donors, CF lung macrophages exhibit alternative methylation patterns in genes associated with the phagocytic response to infection [75], while CF nasal epithelial cells display differential methylation in genes involved in the inflammatory response [76]. It is possible that immune reprogramming of the host by early-in-life bacterial infections through trained immunity contributes to the establishment of chronic infections by decreasing the host immune response to infection over time.

**Figure 3:**
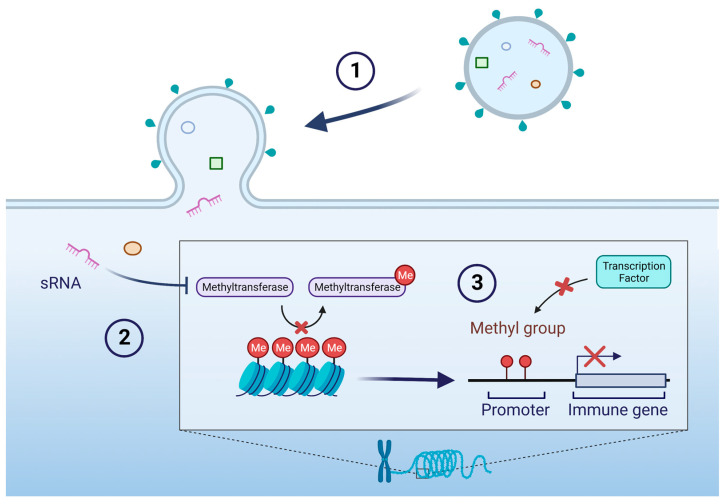
Example of epigenetic regulation of host genes by sRNA delivered by OMV. (**1**) OMV fuses with a host cell and delivers contents including an sRNA. (**2**) The sRNA inhibits a methyltransferase, thereby preserving methyl groups on chromosomal DNA. (**3**) The presence of methyl groups obstructs the binding of transcription factors to promoter regions of immune genes, resulting in reduced expression of immune gene transcripts. Additional epigenetic mechanisms include modulating the expression of DNA methylation modifiers, such as ten-eleven translocation (TET) methylcytosine dioxygenases and DNA methyltransferases (DNMT) [84].

Trained immunity is mediated by epigenetic modifications, which are heritable changes to the DNA and chromatin structure that regulate gene expression [69,70,71]. These epigenetic modifications take the form of the alternative methylation of immune genes and their upstream transcriptional regulators. Over 70% of promoters located at a gene’s transcription start site contain dense cytosine regions that precede a guanine nucleotide (CpG sites) [77]. Methylation of these regions can recruit proteins involved in the repression of genes or inhibit transcription factors from binding DNA [78]. Another critical mechanism is histone modification, wherein chemical groups such as acetyl, methyl, or phosphate are added or removed from histone proteins around which DNA is wrapped [79]. These modifications alter the structure of chromatin and thereby alter the availability of promoter regions, enhancing either gene activation or repression [79]. Exposure to certain stimuli can cause these modifications in immune and epithelial cells, leading to changes in the expression of genes involved in immune function and metabolism (Figure 3) [71,80]. This can result in an enhanced or suppressed immune response to subsequent infections, as well as alterations in other physiological processes such as metabolism and inflammation [80]. The concept of trained immunity was first proposed in the context of vaccination, where it was observed that inoculation with a certain pathogen could provide protection against multiple pathogens beyond the specific target [70,81]. This was thought to be due to the training of the immune system to respond more effectively to subsequent infections. However, bacteria can take advantage of this system for their own benefit, delivering factors to the host that encourage a downregulation of the immune response to infection in order to establish chronic infections. In a proof-of-concept study, exposure of human bronchial epithelial cells to *P. aeruginosa* flagellin decreased epithelial cell secretion of IL-8 upon secondary treatment with LPS, *P. aeruginosa*, *Aspergillus fumigatus*, or *Stenotrophomonas maltophilia* [82]. This effect was lost upon treatment with compounds that inhibit either histone acetyltransferase or histone methyltransferase, suggesting that epigenetic mechanisms are involved in reprogramming the transcriptional immune response [82]. Epigenetic reprogramming by bacterial infections extends beyond in vitro studies and has been demonstrated in an in vivo murine infection model. Brindisi et al. reported a significant reduction in proinflammatory cytokines in the CF mouse lung following *P. aeruginosa* infection, achieved by using a chemical inhibitor that targets histone deacetylase 6 (HDAC6), a major player in CF proinflammatory phenotype dysregulation [83]. By inhibiting HDAC6 activity and consequently increasing histone acetylation, the authors successfully demonstrated a notable reduction in interleukins and chemokines involved in the proinflammatory response to infection [83]. More research has shown that trained immunity can be induced by non-specific stimuli, such as exposure to microbial components like OMVs [22,23].

OMVs also play a significant role in suppressing the host immune response in CF, where chronic bacterial infections are established in a thick mucus layer in the lungs overlying epithelial cells, limiting direct contact of the invading pathogens with host lung epithelia [85]. As several bacterial virulence pathways require direct contact with the host—for example, the Type III secretion system—these systems are unlikely to be relevant if the bacteria and host cells are not in contact. Recent studies have linked initial exposure to OMVs to induce alterations in immune responses following subsequent infection [2,29,46,86]. For example, OMVs secreted by the anaerobic Gram-negative pathogen *Porphyromonas gingivalis* mediate LPS tolerance to subsequent infections of *P. gingivalis* or *E. coli* LPS through the inhibition of pro-inflammatory TNFα and IL1-β secretion [87,88]. An sRNA in *P. aeruginosa* OMVs decreases the LPS-stimulated IL-8 response in human bronchial epithelial cells and diminishes the secretion of the mouse keratinocyte-derived chemokine (KC, the mouse homologue of IL-8), and infiltration of neutrophils in the lung in an in vivo murine model [2]. It is possible, although not yet tested, that this effect of OMVs is due to changes in chromatin accessibility or DNAm mediated by sRNAs. A few recent studies have linked bacterial OMVs to changes in methylation of immune cell genes, altering their response to infection. For example, *B. thetaiotaomicron* OMVs ameliorated chronic intestinal inflammation in a mouse model by increasing methylation of the 4th lysine residue of the histone H3 protein in murine bone-marrow-derived macrophages [23]. On the other hand, treatment with OMVs secreted by *P. aeruginosa* caused a decrease in the methylation of CpG sites in human lung macrophages, which had a strong negative correlation with pro-inflammatory immune cytokine gene expression [22]. Han et al. conducted a comprehensive review focusing on OMVs in respiratory diseases. Their review not only explored the biology of OMVs, but also delved into the signaling pathways that could potentially undergo epigenetic modifications, thereby influencing the immune response to bacterial infections [84]. Although only a handful of studies have examined the mechanisms underlying OMV-induced epigenetic modifications, this is an emerging field within OMV biology. Further research is encouraged on specific epigenetic changes caused by contact with OMVs.

## 5. OMV Characterization

To ensure that the results of OMV research are reliable and meaningful, rigorous and reproducible methods are essential. A critical aspect of rigor is the use of standardized protocols for the isolation, purification, and characterization of OMVs. This can help to ensure that the samples are consistent and comparable across different studies. The International Society for Extracellular Vesicles (ISEV) has published recommendations for the isolation and characterization of OMVs [89]. According to the ISEV, differential ultracentrifugation is the most common method of EV isolation [89,90]. Techniques such as density gradients, filtration, and immunoisolation, among others, are also commonly used for OMV isolation, and recent papers have suggested that ultracentrifugation alone does not result in the most contaminant-free preparation of OMVs [89,91,92,93,94]. Thus, the ISEV recommends that combining isolation methods is more effective in obtaining relatively contaminant-free OMV preparations than any single method [89]. The ISEV recommendations also include suggestions for both qualitative and quantitative methods of characterization. For example, the ISEV recommends providing images of OMV preparation by either electron microscopy, atomic-force microscopy, or super-resolution microscopy [89]. Quantitative measures such as total protein or lipid quantification, the use of protein markers, and nanoparticle tracking analysis (NTA) are recommended by the ISEV for determining OMV concentration and size [89]. Of these methods, NTA is one of the most commonly used for the characterization of particle size and concentration. However, this method is limited since it measures total particles, including both OMVs and any other non-OMV particles present in an OMV preparation, such as protein and lipoprotein aggregates [95,96]. Due to this, NTA tends to overestimate the OMV number, as it reports the hydrodynamic radius of all particles, both OMVs and non-OMV contaminates. In recent years, significant strides have been made in the field of EV and OMV quantification, offering alternatives to NTA. One such advancement is the implementation of tunable resistive pulse sensing (TRPS), which allows for label-free, real-time detection and sizing of EVs and OMVs. TRPS utilizes nanoscale pores to measure changes in electrical potential caused by the passage of vesicles through the pores, providing valuable information about their size and concentration [97]. A notable challenge associated with TRPS lies in the interference of background noise, particularly with smaller particles around 50 nm in size [98,99]. Progress is being made to improve this characterization method, and Ejjigu et al. have addressed the interference of environmental background noise by designing an external shield specifically tailored to mitigate such noise [99]. Additionally, flow cytometric approaches have been used for the quantification of EVs and OMVs [100,101]. Moreover, vesicle studies should always visualize their vesicle preparations with cryo-EM or transmission electron microscopy (TEM) to obtain additional size measurements to those reported by NTA, as these methods report the size of the densest portion of the OMV membrane and allow researchers to distinguish between OMVs and contaminating particles. According to the guidelines set forth by the ISEV, researchers should characterize vesicles by multiple methods to ensure accurate particle size and count with their EV/OMV preparations [89]. Using multiple, orthogonal approaches to EV/OMV characterization, such as both NTA and EM, will improve the rigor, reproducibility, and validity of results. As of this review, updated guidelines for vesicle isolation and characterization are in preparation by the ISEV.

Characterization of OMVs is especially important to ensure rigor and reproducibility in the field, as various factors can alter OMV production, characteristics, and biological effects on the host. It is important to consider relevant in vivo environments when designing in vitro OMV studies. Recent publications have found bacterial OMVs in human biofluids, such as bronchoalveolar fluid in the lungs, blood and urine [102,103,104,105,106]. Many factors are known to affect OMV content and production, including nutrient availability and growth state. The composition of culture media plays a critical role in OMV production and content in Gram-negative bacteria. Different media formulations, such as rich media or minimal media, can result in variations in carbon sources and nutrient availability, which influence bacterial growth and OMV production, as shown in *Francisella novicida*, *Neisseria meningitidis*, and *Bordetella* spp. [107,108,109,110]. Additionally, growth conditions, such as temperature and pH, can also impact OMV production. Hypervesiculation is a method utilized by bacteria to rid themselves of misfolded proteins in response to environmental stress, such as increased temperature [26]. pH is another environmental factor that alters OMV production and composition. At neutral pH, OMVs produced from *Salmonella enterica* have a lipid A composition that parallels that of the bacterial outer membrane [111]. As acidity in the medium increases, OMVs become larger and less protein-dense than those produced in a neutral pH medium [111]. Thus, researchers should consider isolating OMVs from bacteria grown under conditions that reflect the in vivo environment they wish to model. For example, in recent studies examining the effect of *P. aeruginosa* OMVs on CF airway epithelial cells, *P. aeruginosa* was grown in artificial sputum in anoxic conditions [112,113,114], which is similar in composition to the mucus overlying airway cells in the CF lung [85]. It is also worth noting that biofilm formation, a common mode of bacterial growth in various environments, can significantly affect OMV production. The size of OMVs can be influenced by biofilm formation, attributed to the distinct microenvironment and the necessity for intercellular communication within the biofilm matrix [115]. Although numerous studies have primarily focused on OMVs derived from planktonic cells, comparisons between biofilm-derived and planktonic-derived OMVs have been conducted, albeit without reaching a consensus [115,116,117]. Notably, Cooke et al. observed that biofilm-derived OMVs from *P. aeruginosa* are larger in size when compared to their planktonic counterparts [115], while Johnston et al. reported the opposite, suggesting smaller sizes [116]. Conversely, other studies found no statistically significant difference in either *P. aeruginosa* or *Bordetella pertussis* [117,118]. Despite the inconsistency regarding OMV size, several investigations have demonstrated that biofilm-derived OMVs exhibit a higher DNA content than their planktonic counterparts [115,116,119,120]. These findings highlight the complex interplay between growth conditions and OMV production.

In addition to isolation and characterization methods, careful attention should be paid to the statistical analysis of data and the use of appropriate controls. This includes using uninoculated media run through the OMV isolation process to control for contaminants that may remain in the OMV preparation from the media or isolation process (i.e., processed controls). In addition, if possible, experiments using OMV-depleted media are useful controls. Reproducibility is also critical in OMV research, and efforts should be made to replicate key findings using multiple clinical strains of the bacteria of interest and independent orthogonal isolation methods. In addition, for in vitro experiments, it is recommended to use primary cultures of host cells from multiple male and female donors rather than rely on a single immortalized or tumor cell line that is derived from one donor and may not mimic the phenotype/genotype of primary cells. Experiments involving research on host–pathogen interactions with OMVs should also extend to in vivo models to substantiate previous in vitro findings. Ultimately, by adhering to rigorous and reproducible methods, OMV researchers can improve the validity and impact of their findings in this exciting and rapidly evolving field.

## 6. Conclusions and Future Research Prospects

In conclusion, the interplay between Gram-negative bacteria and host cells mediated by OMVs is a complex process that remains incompletely understood. While significant progress has been made in characterizing the immunomodulatory properties of OMVs, further research is needed to elucidate the underlying mechanisms of these interactions. Specifically, future studies should focus on elucidating the mechanism of OMV generation and differential loading of sRNA, tRNA fragments, virulence factors, and other bacterial components in OMVs. Furthermore, studies should also focus on identifying the specific molecular components of OMVs responsible for inducing epigenetic modifications of host immune genes, as well as investigating the downstream effects of these modifications on host immune responses to subsequent infections. The potential utilization of OMVs to induce a diminished immune response and foster trained tolerance within the host could represent a significant mechanism employed by bacteria to establish persistent infections. This becomes particularly noteworthy in the investigation of conditions marked by prolonged bacterial infections, like cystic fibrosis. A more comprehensive understanding of the mechanisms of OMV biogenesis and cargo selection will facilitate the development of new therapeutic strategies for treating bacterial infections. In addition, mechanistic studies on how proteins and sRNA- and tRNA-fragments regulate gene expression in eukaryotic hosts deserves special attention. Very little is known about how bacterial sRNA- and tRNA-fragments regulate eukaryotic gene expression. Finally, while studies have started to shed light on Gram-positive bacterial vesicles [27], our understanding of them pales in comparison to our knowledge regarding those secreted by Gram-negative species. Therefore, it is crucial for further investigations to delve into the contents of Gram-positive vesicles, their impacts on host cells, and interactions between different bacterial species.

Overall, continued investigation into the mechanisms underlying OMV-mediated host-pathogen interactions using rigorous and reproducible methodology will provide important insights into bacterial pathogenesis and immune modulation and may ultimately lead to the development of novel therapeutic approaches to combat chronic antibiotic-resistant bacterial infections.

## Data Availability

Not applicable.
